# Preconditioning of Human Mesenchymal Stem Cells to Enhance Their Regulation of the Immune Response

**DOI:** 10.1155/2016/3924858

**Published:** 2016-10-16

**Authors:** Arman Saparov, Vyacheslav Ogay, Talgat Nurgozhin, Medet Jumabay, William C. W. Chen

**Affiliations:** ^1^Department of Biomedical Sciences, Nazarbayev University School of Medicine, Astana 010000, Kazakhstan; ^2^Stem Cell Laboratory, National Center for Biotechnology, Astana 010000, Kazakhstan; ^3^Center for Life Sciences, Nazarbayev University, Astana 010000, Kazakhstan; ^4^Division of Cardiology, David Geffen School of Medicine at UCLA, Los Angeles, CA 90095, USA; ^5^Research Laboratory of Electronics and Department of Biological Engineering, Massachusetts Institute of Technology, Cambridge, MA 02139, USA

## Abstract

Mesenchymal stem cells (MSCs) have attracted the attention of researchers and clinicians for their ability to differentiate into a number of cell types, participate in tissue regeneration, and repair the damaged tissues by producing various growth factors and cytokines, as well as their unique immunoprivilege in alloreactive hosts. The immunomodulatory functions of exogenous MSCs have been widely investigated in immune-mediated inflammatory diseases and transplantation research. However, a harsh environment at the site of tissue injury/inflammation with insufficient oxygen supply, abundance of reactive oxygen species, and presence of other harmful molecules that damage the adoptively transferred cells collectively lead to low survival and engraftment of the transferred cells. Preconditioning of MSCs* ex vivo* by hypoxia, inflammatory stimulus, or other factors/conditions prior to their use in therapy is an adaptive strategy that prepares MSCs to survive in the harsh environment and to enhance their regulatory function of the local immune responses. This review focuses on a number of approaches in preconditioning human MSCs with the goal of augmenting their capacity to regulate both innate and adaptive immune responses.

## 1. Introduction

Mesenchymal stem cells (MSCs) are a heterogeneous fibroblast-like population of cells that can be isolated from nearly all human tissues, including, but not limited to, bone marrow, adipose, skeletal muscle, heart, umbilical cord, and placenta. MSCs have attracted the attention of scientists and clinicians due to their multilineage differentiation potential and active participation in tissue repair and regeneration after migration to the site of tissue injury [1–3]. When stimulated by appropriate signals, MSCs are capable of differentiating into a number of specialized cell types such as adipocytes, osteoblasts, chondrocytes, and, less frequently, endothelial cells and cardiomyocytes [4–6]. In addition, MSCs possess strong immunosuppressive and immunomodulatory properties that are mediated by both cell-cell contact and production of various signaling factors. MSCs are capable of suppressing or modulating the components of the innate immune system and the functions of T and B cells including regulatory cells, along with influencing Th1 and Th2 cell differentiation [7–10]. Another advantage of MSCs is their immunoprivilege and relative safety when used in allogeneic hosts. However, they can lose their immunoprivilege after differentiation by upregulating MHC class II and Ia expressions and downregulating the expression of immunosuppressive MHC class Ib [7]. Moreover, the reduction in prostaglandin E2 production plays a role in losing MSC hypoimmunogenicity. Despite the large number of published data regarding the benefits of MSCs in preclinical experimental settings, the use of MSCs in treating patients with various immune system related diseases showed mixed results [11, 12]. This discrepancy is presumably attributable to the inability of MSCs to survive and/or to function properly in the inhospitable host environment.

One of the approaches to improve the ability of MSCs to survive in the harsh microenvironment and to enhance their regulation of the immune responses is to precondition the cells* ex vivo* in the specifically designed/engineered environment with different physical or chemical parameter(s)/factor(s). For example, hypoxic preconditioning and three-dimensional culture aggregates are common approaches to mimic natural MSC habitats in tissues, such as bone marrow and adipose tissue, for enhancing MSC survival. The exposure of MSCs to hypoxia caused AKT and BAD phosphorylation as well as the increase in BAG1 and BCL-XL mRNA expression and, as a result, improved cell survival by activating antiapoptotic signaling mechanisms [13]. Hsiao and colleagues reported that hypoxia preconditioned MSCs increased their survival by ATK activation and enhanced their migration by upregulating chemokine receptors, such as CXCR4 and CX3CR1 [14]. Increased survival and migration of MSCs exposed to hypoxia* ex vivo* could explain the increased tissue regenerative potential of these cells in the hind limb ischemia injury model [15]. Beegle and colleagues demonstrated that hypoxic preconditioning of MSCs increased their survival by reducing glucose consumption and lactate secretion as well as lowering the levels of cytochrome c and heme oxygenase 1 [16]. The combination of hypoxia with reoxygenation further increased the expression of prosurvival proteins (HIF-1*α*, erythropoietin receptor, and phosphorylated AKT) and trophic factors (VEGF, ANG, FGF, and BDNF) by MSCs [17]. Furthermore, MSC cultures in three-dimensional aggregates increased their survival by reducing caspase 3/7 activity [18, 19] and by upregulating BCL-2 and AKT [20].

Taking into consideration the natural interactions between immune cells and MSCs at the site of tissue injury/inflammation [8, 21, 22], it is important to enhance the ability of MSCs to regulate the immune response. For example, if MSCs can effectively regulate the function of inflammatory cells that actively damage the surrounding tissue and/or participate in cell debris clearance at the site of injury, this would be likely to enhance tissue healing, which ultimately reduces scar formation. To enable the dampening of the acute inflammatory response and to promote the regeneration of the functional tissue, it is important to enhance the capacity of MSCs to induce constructive immune response after injury. This review focuses on various approaches in preconditioning human MSCs with the goal of enhancing their ability to regulate both innate and adaptive immune responses.

## 2. The Effects of Preconditioned Human Mesenchymal Stem Cells on the Innate Immune Response

MSCs are capable of influencing the function of various cells and components of innate immunity including monocytes/macrophages, neutrophils, natural killer (NK) cells, and the complement system [23, 24]. This section discusses recently published data on the effect of MSC preconditioning on the regulation of the innate immune response.

### 2.1. Hypoxia-Treated MSCs and Innate Immunity

When exposed to hypoxia, MSCs upregulated expression of antiapoptotic proteins to improve their survival in the hostile environment and thus to increase availability of locally functional MSCs [13–16]. Hypoxia also significantly increased the proliferation of bone marrow-derived MSCs (BM-MSCs) and production of IL-6 and IL-8 by the treated cells [25]. Moreover, the conditioned media from hypoxia-treated MSCs increased migration of CD14+ monocytes* in vitro* and macrophages* in vivo* in an experimental model of wound healing [25].

### 2.2. Treatment of MSCs with Cytokines and Innate Immune Response

A number of studies report the effects of cytokine-preconditioned MSCs on innate immunity [26–29]. Noone and colleagues demonstrated that the pretreatment of human MSCs with interferon gamma (IFN-*γ*) results in increased suppression of NK activation and enhanced protection of MSCs from NK-mediated cytotoxicity [26]. This was in part through upregulation of indoleamine 2,3-dioxygenase (IDO) and prostaglandin E-2 (PGE-2) synthesis. In addition, INF-*γ* pretreated MSCs increased expression of nonclassical MHC ligands for the NK inhibitory receptor CD94/NKG2A and reduced expression of activating ligands ULBP1–3 which is recognized by NKG2D [26]. Combining IFN-*γ* treatment with TNF-*α* significantly increased the ability of MSCs to secrete factor H, which is a key molecule involved in the inhibition of complement activation [30]. The depletion of factor H in the MSC-conditioned serum-free media abolished their complement inhibitory activities. Moreover, the inhibition of IDO and PGE-2 production decreased the production of factor H by MSCs, which indicates an important role of these molecules in the production of factor H [30]. Furthermore, François and colleagues reported that the treatment of BM-MSCs with TNF-*α* and IFN-*γ* increased the production of IDO, which participated in the differentiation of CD14+ monocytes into IL-10 producing anti-inflammatory CD206+ M2 macrophages [27]. Moreover, the generated anti-inflammatory M2 macrophages were capable of suppressing the proliferation of human peripheral blood mononuclear cells that were activated via CD3/CD28 pathway [27].

The treatment of umbilical cord-derived MSCs (UC-MSCs) with IL-1*β* significantly upregulated the expression of cyclooxygenase-2 (COX-2), IL-6, and IL-8 compared with untreated MSCs [28]. IL-1*β* treatment also enhanced the MSC production of CXCR4 and the ability of MSCs to migrate* in vitro* [28]. In addition, IL-1*β*-treated MSCs improved their ability to migrate to the spleen, mesenteric lymph nodes, and colon in the experimental model of colitis in mice and contributed to the reduction of the number of M1 macrophages in the murine peritoneal cavity. Using a similar treatment, Carrero and colleagues reported that IL-1*β* preconditioning of MSCs isolated from bone marrow increased expression of multiple cytokines including TNF-*α*, IL-6, IL-8, and IL-23A and chemokines such as CCL5, CCL20, CXCL1, CXCL3, CXCL5, CXCL6, CXCL10, and CXCL11 as well as adhesion molecules VCAM-1, ICAM-1, and ICAM-4 [29]. Furthermore, the treatment of MSCs with IL-1*β* also increased their ability to recruit neutrophils, monocytes, lymphocytes, and eosinophils, with NF-*κ*B playing an important role in this process [29]. These data indicate that the pretreatment of MSCs with IL-1*β* not only increases expression of multiple cell adhesion molecules, which in turn facilitate the migration of MSCs to the site of injury/inflammation, but also enhances the production of numerous chemokines, which are capable of recruiting immune cells and cytokines, which can regulate the function of the target cells.

### 2.3. Treatment of MSCs with TLR Ligands and Innate Immunity

Conditioned media from LPS-treated human UC-MSCs significantly increased the expression of anti-inflammatory cytokines, including IL-10 and TGF-*β*, and reduced expression of proinflammatory cytokines, such as IL-1, IL-6, and TNF-*α*, by human monocytic cell line. The conditioned media also increased the monocyte's preferential differentiation towards anti-inflammatory M2 macrophages [31]. This effect may be mediated by let-7 microRNA through the TLR4/NF-*κ*B/STAT3/ATK signaling pathway. In addition, let-7 microRNA participate in the differentiation and effector function of natural killer T cells [32].

Recently, Fuenzalida and colleagues compared the stimulation of UC-MSCs via toll-like receptor 4 (TLR4) by using LPS, a TLR4 ligand, to the stimulation via TLR3 by using polyinosinic-polycytidylic acid (poly(I:C)), a TLR3 ligand [33]. The treatment of MSCs with poly(I:C) significantly increased expression of IDO compared to the untreated cells, while LPS treatment showed no effect on the MSC IDO expression. However, both poly(I:C) and LPS elevated the levels of IL-6 and IL-8 in MSCs compared to the untreated cells; LPS treatment demonstrated higher expression of both cytokines. These results suggest that MSCs stimulated via TLR3 have a stronger immunosuppressive phenotype with IDO upregulation compared to MSCs stimulated via TLR4.

PGE-2 is an important molecule that converts proinflammatory M1 macrophages into anti-inflammatory M2 macrophages. Gray and colleagues screened several molecules, including IFN-*β*, IFN-*γ*, IL-1*β*, IL-6, LPS, and poly(I:C), on their ability to enhance the immunomodulatory properties of MSCs [34]. IFN-*β*, IFN-*γ*, IL-1*β*, or LPS treatment of MSCs significantly increased the production of PGE-2, with IL-1*β* and LPS having the highest impact. The pairing of these factors demonstrated that the combination of IL-1*β* and LPS had the greatest effect on the production of PGE-2. The downregulation of PGE-2 production in macrophages by MSCs may be mediated by the inhibition of MAPK and ERK phosphorylation [35]. In addition, the treatment of MSCs with TGF-*β* can also decrease the production of PGE-2 by MSCs and decrease the number of CD14+ CD206+ macrophages [36]. Moreover, the assessment of TNF-*α* production, as an important proinflammatory cytokine produced by macrophages, showed that pretreatment of MSCs with IL-1*β* or LPS had the greatest impact on the suppression of TNF-*α* production by human macrophages. However, the combination of these two factors reduced the ability of MSCs for suppression [34]. Thus, these data demonstrate that the combination of two most effective factors can unexpectedly decrease MSCs' ability to regulate the immune response compared to the use of a single factor. This suggests potential conflicts in regulatory signals.

### 2.4. 3-Dimensional Cell Culture of MSCs and Innate Immune Response

In addition to using various factors and hypoxia, a 3-dimensional cell culture system has been used in order to mimic* in vivo* development and to improve the immunomodulatory properties of human MSCs. For example, it has been shown that conditioned medium from MSC spheroids more effectively inhibited the production of TNF-*α*, IL-6, IL-12p40, IL-23, and CXCL2 and increased the production of anti-inflammatory cytokines IL-1ra and IL-10 by LPS stimulated macrophages. MSC spheroid conditioned medium also induced higher production of PGE-2 in LPS stimulated macrophages when compared to the conditioned medium from the MSC monolayer culture counterpart [37]. In addition, the formation of spheres by MSCs leads to self-activation of tumor necrosis factor alpha-induced protein 6 (TSG6) and COX-2 that increase PGE-2 production and conversion of proinflammatory M1 macrophages into anti-inflammatory M2 macrophages, as well as more effectively suppressing TNF-*α* production by LPS stimulated macrophages [38, 39]. In another study, it was demonstrated that the immunosuppressive effect of MSC spheroids on the functional activity of macrophages can be significantly increased by the combinatorial treatment of IFN-*γ* and TNF-*α* [40]. In particular, MSC spheroids preconditioned with IFN-*γ* and TNF-*α* enhanced the suppression of TNF-*α* production by macrophages compared to the untreated spheroids. However, it was observed that immunomodulatory factor secretion is highly dependent on the composition of the cell culture medium. Human MSC spheroids in MesenCult-XF serum-free medium displayed significantly less expression of PGE-2, IDO, TGF-*β*1, and IL-6 compared to MSCs cultured in the same medium supplemented with fetal bovine serum [40]. Moreover, Ylostalo and colleagues have also demonstrated that MSC activation in a 3D environment critically depends on the culture medium. They revealed that the addition of human serum albumin into defined xeno-free culture media resulted in compact MSC spheres with high cell viability, together with high expression of anti-inflammatory molecules (PGE-2 and TSG6). Moreover, it was found that MSC spheres cultured in this medium markedly inhibit the activity of LPS stimulated macrophages [41].

Thus, there are multiple MSC preconditioning approaches that can be utilized either singly or combinatorically to enhance MSC regulation of the innate immune system.  Table 1 summarizes these approaches in MSC preconditioning and their effects on the innate immune response.

## 3. The Effects of Preconditioned Human Mesenchymal Stem Cells on the Adaptive Immune Response

An important feature of MSCs is their powerful immunomodulatory effect on the adaptive immune system, which makes them attractive cellular therapeutic agents to treat inflammatory diseases, such as graft versus host disease, systemic lupus erythematosus, and rheumatoid arthritis and to maintain peripheral immunological tolerance after organ transplantation [42–48]. Over the past decade, a number of studies reported that preconditioning of MSCs promotes their immunomodulatory effects on the cells of the adaptive immune system [49]. To improve the therapeutic efficacy of MSCs, different preconditioning strategies have been adopted. Hypoxic stimulation and cytokine application are two major approaches used for the optimization of the immunomodulatory properties of MSCs.

### 3.1. Hypoxia-Treated MSCs and Adaptive Immunity

For the preconditioning of MSCs, hypoxic pretreatment enhances the immunomodulatory effect of MSCs by improving the secretion of cytokines and soluble factors related to immunosuppression [50–52]. For example, it has been reported that hypoxia modulates the cytokine profile of human gingiva-derived MSCs (G-MSCs), by stimulating the production of anti-inflammatory cytokines, such as IL-10, and shifting the cytokine profile towards the anti-inflammatory mediators. In one study, hypoxia promoted the immunosuppressive effects of human MSCs by increasing the IL-10 production and FasL expression by human G-MSCs, thereby enhancing their prohibitory effect on peripheral blood mononuclear cells (PBMCs) proliferation and promoting the induction of PBMC apoptosis [53]. Roemeling-Van Rhijn and colleagues showed that hypoxia markedly upregulated expression of IDO in human adipose tissue-derived MSCs (AT-MSCs) [54], which is critical in immune regulation by MSCs in part through induction of T cell anergy, generation of regulatory T cells (Tregs), and increased IL-10 production by effector T cells [55, 56]. PGE-2 also plays an important role in mediating the suppression of T cell proliferation by human mesoangioblasts [56]. Additionally, it was demonstrated that coculture of AT-MSCs in hypoxic condition enhanced the suppression of CD4+ and CD8+ T cell [54] as well as PBMC proliferation [57] in comparison to normoxic culture in both cell-cell contact and transwell conditions.

### 3.2. Treatment of MSCs with Cytokines and Adaptive Immune Response

In addition to hypoxic preconditioning, MSCs have the ability to inhibit immune cell activity in response to inflammatory cytokines and soluble factors. This process, termed priming, is very complex and little is known about all of the factors and signaling pathways involved [24]. One of the most studied mechanisms by which inflammation triggers MSC immunomodulatory activity is a treatment with IFN-*γ*. Krampera and colleagues demonstrated an important role of IFN-*γ*, which is mainly produced by NK, CD4+, and CD8+ T cells, in the regulation of immunomodulatory functions of MSCs [58]. The immunosuppressive activity of MSCs on proliferation of T and NK cells is partially mediated by the enhancement of IDO production. This data shows that the inflammatory environment stimulates the function of MSCs and the blockade of IFN-*γ* abrogates this effect. Moreover, it was reported that IFN-*γ*-treated human BM-MSCs markedly suppress Th1 cytokine (IFN-*γ*, TNF-*α*, and IL-2) production by T cells and their proliferation [59]. Although IFN-*γ*-treated MSCs upregulate their IDO expression, the inhibitory function of MSCs can be mediated by B7H1 and B7DC pathways. In another study, it was demonstrated that the treatment of UC-MSCs with IFN-*γ* enhanced their ability to induce a greater number of Tregs in comparison to UC-MSCs not treated with IFN-*γ* [60]. Similar observations by Fan and colleagues demonstrated that IL-1*β*-treated human UC-MSCs significantly increased the numbers of Tregs and Th2 cells, in addition to the decreased numbers of Th1 and Th17 cells in their spleens and mesenteric lymph nodes compared with the control mice in the experimental model of colitis [28].

Recently, it has been reported that the combination of IFN-*γ* and TNF-*α* is more effective for induction of MSCs in expressing high levels of immunosuppressive molecules (IDO, programmed death ligand-1 (PD-L1) and HLA-G) and proinflammatory chemokines including CCL5, CXCL9, CXCL10, and CXCL11 [61]. These chemokines and molecules can induce the accumulation of immune cells in close association with MSCs, thereby forming a microenvironment in which the effects of the locally acting factors produced by MSCs lead to strong immunosuppression [62]. Furthermore, Cuerquis and colleagues demonstrated that the pretreatment of allogeneic MSCs with IFN-*γ* and TNF-*α* significantly increased the suppressive activity of these cells towards CD3/CD28 activated T cells compared to the control group without pretreatment; this effect was mediated by IDO rather than PGE-2 [63]. However, IFN-*γ* and TNF-*α* pretreatment reduced the early surge in IFN-*γ* and IL-2 production by activated PBMCs that was induced by MSCs without any treatment. In regard to unstimulated PBMCs, the pretreatment of MSCs with IFN-*γ* and TNF-*α* increased CD69 expression by CD4+ T cells when compared to the group without any treatment. Moreover, it was shown that INF-*γ* and TNF-*α* promote the ability of human MSCs to express programmed death ligand-2 (PD-L2) and induce the differentiation of CD4+ IL-10+ and CD8+ IL-10+ Treg subpopulations [64].

Prasanna and colleagues directly compared two populations of MSCs isolated either from bone marrow or from Wharton's jelly and reported that pretreatment of BM-MSCs with either IFN-*γ* or TNF-*α* enhanced their ability to suppress mitogen-induced proliferation of lymphocytes while not affecting Wharton's jelly-derived MSCs (WJ-MSCs) [65]. However, the treatment of WJ-MSCs with either IFN-*γ* or TNF-*α* had a more drastic suppressive effect on lymphocyte proliferation in the mixed lymphocyte reaction (MLR) compared to BM-MSCs. These data demonstrate that similar treatment of MSCs isolated from different anatomical locations had different effects on the mitogenic or allogenic T cell responses.

Preconditioning of UC-MSCs with poly(I:C) significantly increased the suppression of T cell proliferation in the MLR compared with untreated cells or MSCs treated with LPS [33]. This result demonstrates differences in the MSC responses to the stimulation of TLR3 or TLR4 ligands; MSCs preconditioned with TLR3 ligands produced a stronger immunosuppressive phenotype compared with MSCs preconditioned with TLR4 ligands.

In addition to the treatment of MSCs with IFN-*γ*, TNF-*α*, and IL-1*β* for the enhancement of their regulation of the immune response, a novel approach that uses IL-17A was recently reported as an alternative approach for cell preconditioning. Sivanathan and colleagues demonstrated that IL-17A treatment did not upregulate either MHC class I or class II expression by MSCs and more effectively increased the suppression of mitogen-activated CD3+ T cells compared to the IFN-*γ*, TNF-*α*, or IL-1*β* treatment [66]. IL-17A treatment of MSCs further increased their ability to downregulate CD25 expression by the mitogen-activated CD4+ T cells and inhibited Th1 cytokine production (IFN-*γ*, TNF-*α*, and IL-2) by this population of cells. Moreover, IL-17A-treated MSCs, similar to IFN-*γ*-treated MSCs, are capable of increasing or inducing CD4+CD25^high^CD127^low^FoxP3+ Tregs in cocultures with mitogen-activated CD4+ CD25− T cells in a cell-contact dependent manner. These results suggest that IL-17A-treated MSCs increase the generation of Tregs that are potent suppressors T cell activation without upregulation of either MHC class I or class II expression. This promising approach can be further explored in the tolerance maintained after organ transplantation and the treatment of autoimmune diseases.

Other than the common mediators used by MSCs to modulate the immune response such as IDO, PGE-2, and HLA-G, galectin-9 has been shown to be upregulated by MSCs in response to a number of stimuli including IFN-*γ*, TNF-*α*, IL-1a, IL-1*β*, PGE-2, and some TLR ligands [67]. The importance of galectin-9 in the suppression of proliferation of both CD4+ and CD8+ T cells by MSCs was demonstrated by the deletion of galectin-9 gene that reduced the suppressive effects of MSCs. Moreover, galectin-9 plays an important role in the generation and function of inducible regulatory T cells [68].

### 3.3. Glucocorticoid-Treated MSCs and Adaptive Immunity

The use of glucocorticoid steroids is another approach to precondition the cells. Ankrum and colleagues demonstrated that glucocorticoids, such as budesonide and dexamethasone, increased IDO activity in primary human MSCs without altering their metabolic activity, morphology, or viability and upregulating MCH class II [69]. This pretreatment even decreased the expression of MHC class I by PBMC, which can be beneficial when allogeneic MSCs are used in the therapy. Interestingly, the addition of IFN-*γ* to the preconditioned media upregulated the expression of both MHC class I and MHC class II molecules. The increased IDO activity is mediated through glucocorticoid receptor and FOXO3, since the inhibition of either of these molecules abrogates this effect.

Table 2 summarizes the effects of MSCs preconditioning on their ability to regulate the adaptive immune response. In summary, many studies have indicated that preconditioning of human MSCs can be used to enhance their regulation of the adaptive immune response. This approach is definitely worth investigating further for the treatment of autoimmune diseases, particularly for the induction of immune tolerance.

## 4. Conclusions

MSCs have the unique ability of differentiating into a number of cell types and contributing to tissue regeneration after being adoptively transferred. However, a hostile environment at the site of tissue injury leads to a low survival rate and engraftment of the transferred cells [2, 70, 71]. Preconditioning of MSCs by hypoxia, inflammatory stimulus, and other factors/conditions prior to their use in therapy is a new strategy to prepare MSCs to survive and function better after transplantation and to enhance their regulation of the local or systemic immune response. A number of approaches have been proposed to precondition MSCs, including hypoxia, cytokines (IFN-*γ*, TNF-*α*, and IL-1*β*), TLR ligands (LPS and poly(I:C)), and 3-dimensional cell culture. These models demonstrated the possibility of enhancing the regulation of both the innate and adaptive immune responses. Optimization of signaling factors and their combinations to be used in MSC preconditioning should be further investigated. Thus, MSC preconditioning enhances the ability of MSCs to survive in the harsh environment and to regulate both innate and adaptive immune responses and this approach can be further explored in the treatment of patients affected by immune system related diseases.

## Figures and Tables

**Table 1:** Regulation of the innate immune response by preconditioned human mesenchymal stem cells.

Preconditioning approach	The source of cells	Secreted factors or expressed genes	Immunomodulatory effects	Reference
Hypoxia	BM-MSCs	↑ IL-6 and IL-8	↑ monocyte migration *in vitro* and macrophages *in vivo*	[25]
IFN-*γ*	UC-MSCs	↑ IDO and PGE-2	↓ NK activation and protection from NK cytotoxicity	[26]
IFN-*γ* and TNF-*α*	MSCs	↑ Factor H	↓ complement activation	[30]
IFN-*γ* and TNF-*α*	BM-MSCs	↑ IDO	↑ generation of M2 macrophages	[27]
IL-1*β*	UC-MSCs	↑ COX-2, IL-6, and IL-8	↓ number of M1 macrophages	[28]
IL-1*β*	BM-MSCs	↑ TNF-*α*, IL-6, IL-8, and IL-23A, CCL5, CCL20, CXCL1, CXCL3, CXCL5, CXCL6, CXCL10, and CXCL11, and VCAM-1, ICAM-1, and ICAM-4	↑ recruitment of neutrophils, monocytes, lymphocytes, and eosinophils	[29]
LPS	UC-MSCs	↑ IL-10, TGF-*β*, and ↓ IL-1, IL-6, and TNF-*α*	↑ generation of M2 macrophages	[31]
LPS and IL-1*β*	BM-MSCs	↑ PGE-2	↑ generation of M2 macrophages	[34]
TGF-*β*	Decidual MSCs	↓ PGE-2	↓ number of CD14+ CD206+ macrophages	[36]
3D culture	BM-MSCs	↑ COX-2 and PGE-2	↑ generation of M2 macrophages and ↓ TNF-*α* production by LPS stimulated macrophages	[37–39]
IFN-*γ* and TNF-*α* in 3D culture	BM-MSCs	↑ IDO and IL-6	↑ suppression of TNF-*α* production by LPS stimulated macrophages	[40]

**Table 2:** Regulation of the adaptive immune response by preconditioned human mesenchymal stem cells.

Preconditioning approach	The source of cells	Secreted factors or expressed genes	Immunomodulatory effects	Reference
Hypoxia	G-MSCs	↑ IL-10 and FasL	↓ PBMC proliferation and ↑ apoptosis of PBMC	[53]
Hypoxia	AT-MSCs	↑ IDO, PD-L1, and CXCL10	↓ PBMC, CD4+, and CD8+ T cell proliferation	[54, 57]
IFN-*γ*	BM-MSCs	↑ IDO	↓ T cell proliferation, degranulation, and Th1 cytokine (IFN-*γ*, TNF-*α*, and IL-2) production	[59]
IFN-*γ*	UC-MSCs	↑ IDO	↑ expansion of Tregs	[60]
IL1-*β*	UC-MSCs	↑ COX-2, IL-6, and IL-8	↑ Tregs and Th2 cell proliferation and ↓ numbers of Th1 and Th17 cells	[28]
IFN-*γ* and TNF-*α*	BM-MSCs	↑ IDO	↓ T cell proliferation	[63]
IFN-*γ* and TNF-*α*	Placenta-derived MSCs	↑ PD-L2	↑ differentiation of CD4+IL-10+ and CD8+IL19+ Treg subpopulations	[64]
IFN-*γ* or TNF-*α*	WJ-MSCs	↑ IDO, PGE-2, CIITA, and ↓ HGF	↓ lymphocyte proliferation in the MLR	[65]
IFN-*γ* or TNF-*α*	BM-MSCs	↑ IDO, PGE-2, and CIITA	↓ mitogen-induced proliferation of lymphocytes	[65]
IFN-*γ*, TNF-*α*, IL-1*α*, IL-1*β*, and TLR ligands	BM-MSCs	↑ galectin 9	↓ T cell proliferation	[67]
IL17A	BM-MSCs	↑ IDO	↓ T cell activation and proliferation and ↑ expansion of Tregs	[66]
Budesonide	BM-MSCs	↑ IDO	↓ T cell activation and proliferation	[69]
